# Potentially avoidable computed tomography after positive ultrasonography in appendicitis

**DOI:** 10.1590/1806-9282.20260877

**Published:** 2026-10-23

**Authors:** Serdar Derya, İpek Ergün, Ahmet Arda Altuner, Ahmet Ege Büyükfirat, Ahsen Öztürk, Ali Efe Aktürk, Aram Miran Yaruk, Ahmet Deniz Topal, Yağmur Irmak, Zehra Keser

**Affiliations:** 1 Inonu University, Faculty of Medicine, Department of Emergency Medicine – Malatya, Türkiye.; 2 Inonu University, Faculty of Medicine – Malatya, Türkiye.

**Keywords:** Appendicitis, Emergency service, Radiation dosage, Tomography, Ultrasonography

## Abstract

**OBJECTIVE:**

The aim of this study was to determine the frequency of computed tomography performed despite a positive ultrasonography in histopathologically confirmed appendicitis (potentially avoidable computed tomography), and to assess its effects on radiation exposure, cost, and emergency department length of stay.

**METHODS:**

This single-center retrospective cohort study included 887 consecutive patients who underwent appendectomy for suspected appendicitis (January 2023–December 2025). Potentially avoidable computed tomography was defined as computed tomography following a positive ultrasonography result. As length of stay was non-normal, imaging strategies were compared using the Kruskal-Wallis test, and length of stay predictors were modeled using median (quantile) regression, with a log-link gamma generalized linear model as a sensitivity analysis. Imaging cost used national tariffs; radiation was estimated as ~8 mSv per abdominal computed tomography with age-specific BEIR VII multipliers.

**RESULTS:**

Computed tomography was used in 87.6% (777/887) of cases. Of the 521 patients who underwent ultrasonography, the results were positive in 35.5%, inconclusive in 30.7%, and negative in 33.8% of cases. Potentially avoidable computed tomography was performed in 81/777 computed tomography examinations (10.4%). Notably, 43.8% (81/185) of patients with positive ultrasonography results subsequently underwent computed tomography scan, corresponding to ~648 mSv of avoidable radiation exposure and an associated cost of 26,667 TL (~US$811), with 49.4% of the affected patients being children. Length of stay did not differ across strategies (p=0.748), and computed tomography was not an independent length of stay predictor (+7.2 min; p=0.23), whereas business-hour arrival was (+15.3 min; p<0.001).

**CONCLUSION:**

Computed tomography remained frequent even after ultrasonography established the diagnosis, with no effect on emergency department length of stay, generating avoidable radiation and costs concentrated in children. Reducing computed tomography after a positive ultrasonography is a feasible opportunity to improve resource utilization while preserving patient safety.

## INTRODUCTION

Acute appendicitis is one of the most common surgical emergencies worldwide; however, its diagnosis is challenging. Because clinical findings and laboratory tests alone are insufficient, scoring systems combining clinical and imaging data are widely used to support diagnosis and to distinguish complicated from uncomplicated disease^1^. Misdiagnosis and unnecessary appendectomy remain notable, particularly in atypical presentations, and diagnostic delay is associated with perforation and intra-abdominal abscess^2^. Imaging that provides little or no patient benefit lowers care quality and burdens the health system^3^. Reducing low-value imaging is therefore a principal quality-improvement goal in emergency medicine and a core theme of the Choosing Wisely initiative^4,5^.

Ultrasonography (US), being radiation-free and widely available, is the first-line modality, especially in children and non-obese adults^6,7^. However, many US examinations are inconclusive, prompting further imaging or observation^8^. Computed tomography (CT), owing to its high diagnostic accuracy, is an important complementary modality when clinical evaluation is uncertain^9,10^. In some cases, CT is still performed even after US has clearly established appendicitis; we defined such examinations as potentially avoidable CT (PA-CT).

Imaging overuse is a particular focus of Choosing Wisely, which recommends limiting unnecessary or repeated examinations^5^. Abdominal CT delivers about 8 mSv per examination, important in children for lifetime cancer risk; both the ICRP 103 recommendations and the EuroSafe Imaging initiative emphasize keeping dose as low as reasonably achievable (ALARA)^11,12,13^.

Although the diagnostic performance of US and CT is well-defined, studies evaluating how imaging strategies affect resource use and ED operations in histopathologically confirmed cohorts are limited. This is consistent with Chidiac et al., who emphasized reducing radiation exposure in children with suspected appendicitis^14^, and with the systematic review and meta-analysis by Castro-Luna et al. on US, CT, and MRI for acute appendicitis in children and adolescents^15^.

We aimed to determine the frequency of PA-CT in a three-year histopathologically confirmed cohort, its associated radiation burden and cost, and its relationship with ED LOS. Cross-sectional imaging may be clinically necessary to exclude complications or delineate atypical anatomy; therefore, the PA-CT definition reflects potential optimization rather than a direct indicator of care.

## METHODS

### Study design and ethics

This single-center retrospective cohort study was conducted at a tertiary emergency service (January 2023–December 2025), approved by the institutional research ethics committee (no. 2026/9399), and conducted in accordance with the Declaration of Helsinki. The requirement for individual informed consent was waived owing to the retrospective nature of the study. Reporting followed the STROBE statement^16^.

### Participants

All patients presenting with suspected acute appendicitis who underwent emergency appendectomy with complete ED records were included (n=887) and classified by imaging strategy: US only (n=110), US followed by CT (n=411), and direct CT without US (n=366).

### Definitions and variables

A positive US result was defined as a non-compressible, blind-ending tubular structure in the right lower quadrant with an outer diameter >6 mm (with or without wall thickening, periappendiceal fat changes, or an appendicolith). Examinations that did not meet these criteria were classified as negative or inconclusive when the appendix could not be visualized on the images. PA-CT was performed after a positive US result (examinations as potentially avoidable CT). This procedural definition does not include the CT scan requested to rule out appendicitis complications such as suspected perforation, abscess, or retrocecal anatomy. LOS was defined as the interval from triage to the operating room transfer. Imaging costs used national tariffs (CT 329.22 TL; US 78.33 TL), also expressed in US$ (32.9 TL/US$). Radiation was estimated as 8 mSv per abdominal CT^17^ and age-specific BEIR VII risk multipliers^12,13^.

### Statistical analysis

Continuous variables are presented as median (interquartile range), and categorical variables are presented as n (%). As LOS was non-normal (Shapiro-Wilk W=0.82, p<0.001), groups were compared using Kruskal-Wallis and Mann-Whitney tests, and LOS predictors were modeled with median (quantile) regression, with a log-link gamma generalized linear model (GLM) as a sensitivity analysis. Consultation duration was excluded as a within-window subcomponent of LOS. The model included 887 patients after listwise deletion of missing values (mainly leukocyte count, n=56). Categorical comparisons used the χ^2^ or Fisher exact test; baseline comparisons were descriptive. Analyses were performed using Python 3.12 (SciPy, Statsmodels)^18^; p<0.05 was considered significant.

## RESULTS

### Patient characteristics

Of the 887 patients, 57.2% were male and 38.8% were children (<18 years); the median age was 21 years (IQR, 13–41). The negative appendectomy rate was 0.23% (2/887). The baseline characteristics of the patients according to the imaging strategy are presented in Table 1. After Bonferroni correction (p<0.004), differences in age, proportion of pediatric patients, sex, symptom duration, right lower quadrant tenderness, and fever persisted.

**Table 1:** Baseline characteristics by imaging strategy (n=887).

Variable	Overall (n=887)	US only (n=110)	US+CT (n=411)	CT only (n=366)	PA-CT (n=81)	Other CT (n=696)	p-value
Age, years, median (IQR)	21 (13–41)	13 (10–18)	16 (11–31)	33 (22–52)	18 (12–43)	24 (15–44)	<0.001
Pediatric (<18 y), n (%)	344 (38.8)	78 (70.9)	239 (58.2)	27 (7.4)	40 (49.4)	226 (32.5)	<0.001
Male sex, n (%)	507 (57.2)	81 (73.6)	223 (54.3)	203 (55.5)	42 (51.9)	384 (55.2)	<0.001
Symptom duration, h	24 (24–24)	24 (24–25)	24 (15–24)	24 (24–24)	23 (2–24)	24 (24–24)	0.002
RLQ tenderness, n (%)	527 (59.4)	51 (46.4)	219 (53.3)	257 (70.2)	58 (71.6)	418 (60.1)	<0.001
Rebound tenderness, n (%)	188 (21.2)	14 (12.7)	80 (19.5)	94 (25.7)	20 (24.7)	154 (22.1)	0.007
Fever ≥38°C, n (%)	150 (16.9)	32 (29.1)	58 (14.1)	60 (16.4)	8 (9.9)	110 (15.8)	<0.001
WBC, ×10^3^/**μ**L	13.0 (10.5–15.9)	14.0 (11.1–17.9)	13.3 (10.3–16.7)	12.7 (10.5–15.0)	13.4 (10.6–16.6)	13.0 (10.4–15.6)	0.009
Neutrophil%	79.2 (69.7–85.0)	78.0 (70.5–85.1)	80.4 (69.0–86.0)	77.0 (70.0–83.7)	80.0 (72.8–86.0)	79.2 (69.6–84.8)	0.060
CRP, mg/L	2.0 (0.4–6.2)	1.4 (0.1–5.4)	1.6 (0.1–6.8)	2.7 (0.6–6.0)	1.7 (0.5–6.7)	2.0 (0.5–6.2)	0.034
Business-hours arrival, n (%)	439 (49.5)	57 (51.8)	200 (48.7)	182 (49.7)	40 (49.4)	342 (49.1)	0.835
ED LOS, h	5.77 (5.25–6.18)	5.70 (5.30–6.11)	5.75 (5.22–6.17)	5.77 (5.27–6.21)	5.9 (5.4–6.2)	5.8 (5.2–6.2)	0.748
Negative appendectomy, n (%)	2 (0.23)	0 (0.0)	0 (0.0)	2 (0.55)	0 (0.0)	2 (0.3)	0.533

Values are presented as median (IQR) or number (%). p-values from the Kruskal-Wallis test (continuous) and χ^2^ test (categorical), with Fisher’s exact test for expected counts <5. Comparisons were descriptive; under a Bonferroni threshold (p<0.004), age, proportion of pediatric patients, sex, symptom duration, RLQ tenderness, and fever were significant. RLQ: right lower quadrant; LOS: length of stay; WBC: white blood cell; CRP: C-reactive protein; US: ultrasonography; CT: computed tomography; PA-CT: potentially avoidable computed tomography; IQR: interquartile range; ED: emergency department. PA-CT vs. other CT comparisons were post-hoc; under Bonferroni correction (4 tests, threshold p<0.0125), only fever frequency remained significant.

### Imaging strategies and potentially avoidable computed tomography

CT was used in 87.6% of cases (n=777; Figure 1). Of the 521 patients who underwent US, the results were positive in 185 (35.5%), inconclusive in 160 (30.7%), and negative in 176 (33.8%); complementary CT after inconclusive and negative US results was common. PA-CT was performed in 81/777 CT examinations (10.4%); 43.8% of patients nonetheless underwent CT. Compared with other CT patients, PA-CT patients had similar C-reactive protein (1.7 vs. 2.0 mg/L, p=0.829), leukocytes (13.4 vs. 13.0×10^3^/μL, p=0.597), and neutrophils (80.0 vs. 79.2%, p=0.285), and lower fever frequency (9.9 vs. 15.8%, p=0.001). In 772/777 CT examinations, the findings were consistent with the histopathological results. As all patients underwent surgery, the specificity and negative predictive values could not be calculated.

**Figure 1:**
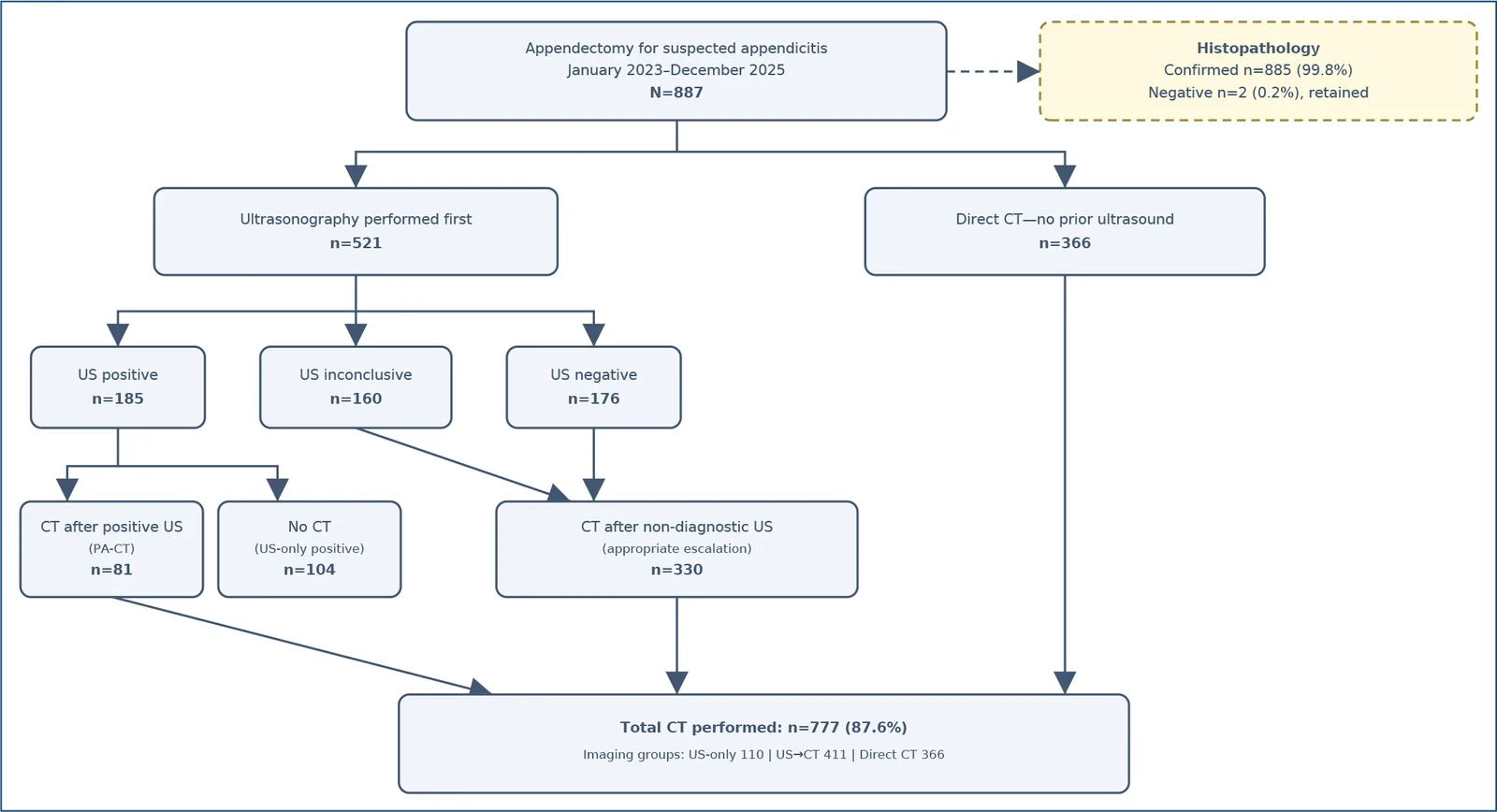
Patient flow and imaging pathway.

### Emergency department length of stay

The median LOS was 5.77 h (IQR 5.25–6.18) and did not differ across strategies (5.70, 5.75, 5.77 h; p=0.748); CT-versus-no-CT (p=0.627) and age group (p=0.444) comparisons were also non-significant. In median regression (n=828), CT was not independently associated with LOS (+7.2 min; 95%CI -4.5 to 18.8; p=0.23), whereas business-hours arrival was (+15.3 min; 95%CI 8.2–22.3; p<0.001); the gamma GLM concurred [CT exp(β)=1.02, p=0.34; business hours exp(β)=1.13, p<0.001]. No clinical or laboratory variable was independently associated with LOS (all p>0.10), and the model’s explanatory power was limited (gamma pseudo-R^2^≈0.09). Among patients with positive US results, the median LOS was 5.88 h with PA-CT versus 5.67 h without CT (p=0.058).

### Cost and radiation

The total imaging cost was 296,614 TL (56.5% from US-guided CT, 40.6% from direct CT). PA-CT alone corresponded to 26,667 TL (~US$811) and 648 mSv of potentially avoidable radiation exposure, respectively. The PA-CT group had a median age of 18 years, and 49.4% of the patients were children (Table 2). The total CT radiation was ~6,216 mSv, of which 2,128 mSv (34.2%) was attributable to the children.

**Table 2:** Imaging cost and radiation by age group.

Age group	CT, n (%)	Total mSv	BEIR VII ×	PA-CT, n	Avoidable mSv	Avoidable cost, TL
0–5 year	27 (3.5)	216	3.0×	4	32	1,317
6–12 year	129 (16.6)	1,032	2.5×	17	136	5,597
13–17 year	110 (14.2)	880	2.0×	19	152	6,255
18–40 year	291 (37.5)	2,328	1.0× (ref)	16	128	5,268
41–60 year	138 (17.8)	1,104	0.6×	18	144	5,926
>60 year	82 (10.6)	656	0.3×	7	56	2,305
Total	777 (100)	6,216	–	81	648	26,667

Avoidable cost=PA-CT count×CT tariff (329.22 TL); total 26,667 TL (≈US$811). CT: computed tomography; PA-CT: potentially avoidable computed tomography; TL: Turkish Lira.

## DISCUSSION

In 887 patients with histopathologically confirmed appendicitis, CT was performed despite a positive US in 10.4% of cases, with 648 mSv of potentially avoidable radiation and ~US$811, with approximately half of the affected patients being children. These post-US CT examinations generated a considerable radiation and cost burden but had no significant effect on ED LOS; the rationale for reducing them therefore relates to preventing unnecessary radiation and using health resources more efficiently rather than to patient flow^19,20^.

The fact that 43.8% of positive US patients still underwent CT may reflect the exclusion of complications, anatomical evaluation for surgical planning, established practice, and medicolegal or defensive medicine considerations, together with limited confidence in operator-dependent US. In multivariable analyses, LOS was independent of the imaging strategy and was driven more by operational factors; business-hour arrival was associated with LOS, whereas CT was not. A longer LOS during business hours likely reflects a higher daytime ED census and competition for imaging and operating room capacity rather than imaging choice and is hypothesis-generating. Additional CT after a positive US provides no measurable advantage to patient flow.

The 35.5% positive US rate may reflect the operator-dependent nature of US and the classification of non-visualized appendices as inconclusive findings. In the positive US subgroup, the trend toward longer LOS in PA-CT patients did not reach significance (p=0.058), consistent with the absence of a clear operational benefit.

Radiation findings are particularly important in children, in whom longer life expectancy and higher radiosensitivity make long-term risks more pronounced than in adults^21,22^. Avoiding unnecessary CT when US adequately supports the diagnosis is therefore feasible for radiation safety, and low-dose CT protocols offer additional risk reduction when CT is needed^9^. A pediatric appendicitis algorithm with a US-first approach and an option for next-day surgery clinic follow-up in uncertain cases reduced CT use from 13.8 to 6%, without increasing US use, hospital admission, surgical consultation, ED return within 7 days, or LOS^23^.

The very low negative appendectomy rate (0.23%) may reflect high imaging use and a selective surgical approach, although histopathological criteria and local practice may also contribute; this should not be generalized to other centers. Importantly, because PA-CT denotes CT performed after an already positive US, it identifies the subset in which CT is least likely to alter the operative decision; therefore, reducing CT in this group is unlikely to compromise the low negative appendectomy rate, which reflects the overall imaging-and-selective-surgery strategy rather than the incremental post-US scan alone.

## LIMITATIONS

PA-CT is a procedural definition. Complicated disease findings (perforation, abscess, plastron) were not systematically recorded; therefore, post-US CT may have been justified in some instances. CT was ordered by both emergency physicians and surgeons; however, the ordering clinician was not recorded in every case, limiting the evaluation of the decision-making process. The retrospective, single-center design and inclusion of only operated patients limit the generalizability of our findings to some extent. Radiation was estimated from reference doses rather than patient-based dosimetry, and the cost analysis covered only direct imaging costs, not indirect costs (equipment depreciation, personnel, and ED crowding). Finally, the models explained only a limited portion of the variation in LOS.

## CONCLUSION

Despite a US-based diagnosis of appendicitis, CT remains frequently used, with no significant effect on ED LOS, and is associated with potentially avoidable radiation and costs concentrated in children. Reconsidering the use of CT after a positive US offers a feasible opportunity to optimize imaging practices. Prospective multicenter studies that capture complicated disease features and clinicians making imaging decisions are needed to confirm these observations and clarify the clinical rationale for post-US CT.

## Data Availability

The datasets generated and/or analyzed during the current study are available from the corresponding author upon reasonable request.
